# Beyond urinalysis: a review of life-course chronic kidney disease screening across health examination systems in Japan

**DOI:** 10.1007/s10157-026-02918-6

**Published:** 2026-07-11

**Authors:** Kimiko Honda, Yoko Fujita, Yugo Shibagaki, Rei Goto, Yoshimitsu Gotoh, Yutaka Harita

**Affiliations:** 1 Graduate School of Health Management, Keio University, Tokyo, Japan; 2 Division of Nephrology and Hypertension, Department of Internal Medicine, St. Marianna University School of Medicine, Kanagawa, Japan; 3 Division of Nephrology and Hypertension, Kawasaki Municipal Tama Hospital, Kanagawa, Japan; 4 Graduate School of Business Administration, Keio University, Tokyo, Japan; 5 Department of Pediatrics, Japanese Red Cross Aichi Medical Center Nagoya Daini Hospital, Aichi, Japan; 6 Department of Pediatrics, The University of Tokyo Hospital, 7-3-1 Hongo, Bunkyo-ku, Tokyo 113-8655, Japan

**Keywords:** Chronic kidney disease, Screening, Urinalysis, Health examination, Life course, Japan

## Abstract

Chronic kidney disease (CKD) is a major public health problem. Most international guidelines recommend targeted testing of individuals at high risk rather than population-wide screening. In Japan, urinalysis is embedded in health examination systems across the life course. However, the existence of repeated urinalysis opportunities has not necessarily meant that these programs functioned as a coherent CKD screening pathway. Repeated urinalyses were introduced under different legal mandates and for different public health purposes, with substantial variation in target conditions, test components, and follow-up arrangements. In this context, the 2025 policy decision to add serum creatinine to workplace-based health examinations, following sustained advocacy by the Japanese Society of Nephrology and growing recognition of non-proteinuric CKD, marks an important policy turning point. This review provides an overview of Japan’s current health examination systems from the perspective of CKD screening throughout the life course. Specifically, this review describes the 3-year-old health examination, school urinary screening, workplace-based health examinations, the Specific Health Checkup, and late-stage elderly medical examinations by focusing on their institutional basis, structure, follow-up pathways, and current limitations. Taken together, Japan’s health examination systems provide repeated urinalyses; however, these opportunities do not automatically amount to a coherent CKD screening strategy. This overview highlights the need to clarify target conditions, downstream interventions, outcome measures, and continuity across programs.

## Introduction

Chronic kidney disease (CKD) affects approximately 700 million people worldwide and often progresses without symptoms until the risks of kidney failure and cardiovascular events are substantial [1]. CKD imposes a substantial economic burden on health systems, with costs increasing sharply as patients progress to kidney failure requiring dialysis or transplantation [2, 3]. Therefore, early detection is a public health priority. Major guidelines recommend targeted testing of individuals at high risk rather than population-wide screening [4, 5].

Japan differs from many other countries in that urinalysis is embedded in health examination systems across the life course, including the 3-year-old health examination, school urinary screening (SUS), workplace-based health examinations, the Specific Health Checkup (SHC), and late-stage elderly medical examinations [6]. However, repeated urinalysis opportunities do not in themselves constitute a coordinated CKD screening pathway. The examinations were introduced for diverse institutional purposes, with different target conditions, test components, and follow-up arrangements. In childhood programs, renal disease detection has been an explicit aim, whereas in adult programs urinalysis has generally been embedded in broader occupational health or lifestyle-related disease frameworks rather than organized primarily around CKD detection. A clear example is the reliance on proteinuria-based urinalysis in adult health examinations, which misses many cases of CKD without overt proteinuria [7]. The Japanese Society of Nephrology (JSN) has described repeated urinalysis opportunities across Japan’s health examination systems as “life-course urinalysis,” while acknowledging that urinalysis alone is insufficient for CKD screening [8, 9]. On that basis, JSN formally requested the addition of serum creatinine to workplace-based health examinations [10].

Supported by growing evidence on occupational risk factors for CKD and by the increasing clinical value of early detection in the era of sodium-glucose cotransporter-2 inhibitors, these longstanding concerns and requests led to a policy change in 2025: serum creatinine was scheduled to be added to workplace-based health examinations, with implementation set to begin in April 2027 [11]. This policy change was also institutionally important because workplace-based health examinations are prioritized over the SHC for adults with employee-based insurance, and their results may be used in place of SHC results [12]. The planned addition of serum creatinine represents a practical starting point for broader reconsideration of CKD screening for adults in Japan.

This reform also highlights the limitations of the current system. Although the 2025 policy change partly addressed this gap, serum creatinine may still be omitted for workers younger than 40 years [13]. Whether 40 years is an appropriate threshold, and whether proteinuria-based testing is the best alternative when serum creatinine is not measured, remain open questions. Equity is another issue: even among adults aged 40 years and older, access to appropriate opportunities for CKD screening is not assured for those not covered by workplace-based health examinations.

The key question, therefore, is whether Japan’s existing health examination systems, taken together, provide an adequate, coherent, and equitable approach to CKD screening across the life course. This review reassesses these health examination systems by focusing on CKD screening and identifies their main gaps in the current system.

## CKD in Japan and urinalysis across the life course

**Fig. 1:**
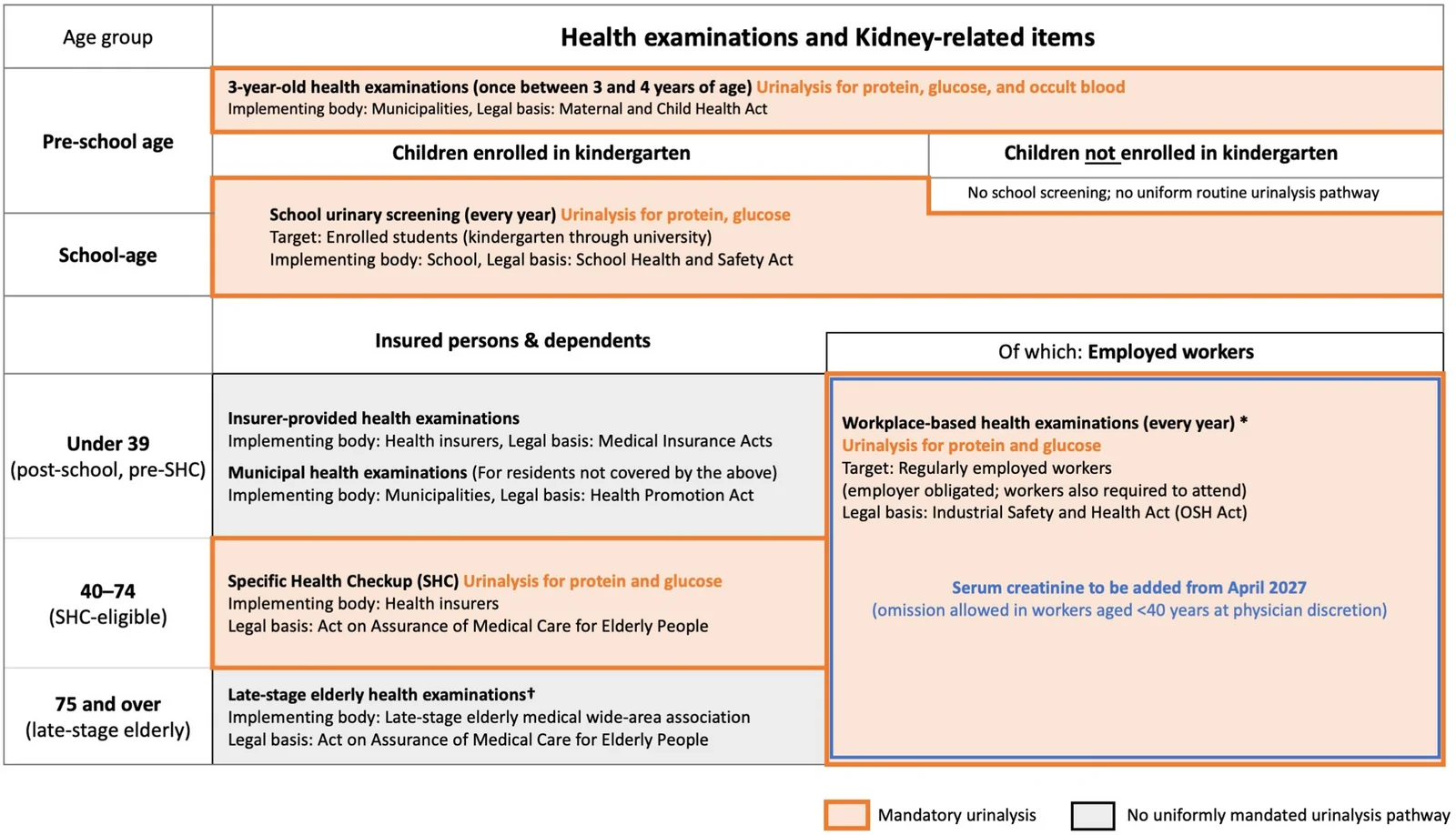
Overview of Japan’s health examination and screening systems and kidney function testing. Adapted from the “Overview of Japan’s Health Examination and Screening Systems” table in materials from 10th Meeting of the Study Group on Examination Items for General Health Examinations under the Industrial Safety and Health Act [64]. *Workplace-based health examinations are operationally prioritized over SHC, and their results may be used in place of SHC results. †For insurer-provided, municipal, and late-stage elderly health examinations, implementation depends on the responsible body on a best-efforts basis rather than a universally mandated pathway. In late-stage elderly health examinations, urinalysis is a basic item when the examination is conducted

CKD imposes a substantial burden in Japan. Adult CKD prevalence was estimated at approximately 13.3 million in 2005 and 14.8 million in 2015 [14]. By the end of 2024, 2,725.4 patients per million population were receiving chronic dialysis and 36,404 patients initiated chronic dialysis in Japan. The causes of kidney failure have also shifted: chronic glomerulonephritis declined from 27.4% in 2005 to 13.5% in 2024, nephrosclerosis increased from 9.0% to 19.1%, and diabetic kidney disease remained the leading cause at 37.6% [15]. Previous studies have suggested that long-standing urinalysis-based screening practices in Japan have contributed to the decline in the incidence of kidney failure caused by glomerulonephritis; however, this possibility was not directly evaluated [16]. This possibility matters for CKD screening because nephrosclerosis and diabetic kidney disease may progress with little or no proteinuria; therefore, urinalysis alone will miss a meaningful number of cases. A unified CKD screening program does not exist in Japan. Instead, urine-based testing is distributed across legally distinct health examination programs for different age groups and public health purposes. Early childhood examinations were developed within maternal and child health services, SUS within the school health system, adult examinations within occupational health and metabolic syndrome prevention programs, and late-stage elderly medical examinations were developed to focus on frailty and lifestyle-related disease. Therefore, repeated opportunities for urinalysis, rather than a coherent screening strategy, exist across the life course, and continuity of kidney-related information across programs is not assured (Fig. 1). A key question is whether these age-specific programs, taken together, provide a coherent pathway to CKD detection, follow-up, and intervention in Japan.

### Early childhood: urinalysis beginning with the 3-year-old health examination

The first component of life-course urinalysis in Japan is the 3-year-old health examination, a municipality-based program within the maternal and child health system [17]. Although primarily intended for developmental surveillance and support for child-rearing, it includes standard urinalysis for protein, glucose, and occult blood [18]. Accordingly, urinalysis at the 3-year-old health examination is best understood as an early population-level opportunity embedded in an existing child health platform, rather than as a kidney-specific screening program. Participation in the 3-year-old health examination was very high, at nearly 100%, whereas urinalysis coverage was lower, in the high 80% range [19, 20].

The clinical rationale for urinalysis at this age has often been discussed in relation to congenital anomalies of the kidney and urinary tract (CAKUT), the leading cause of kidney failure in Japanese children [21, 22]. The Japanese Society for Pediatric Nephrology (JSPN) has regarded this first opportunity for population-based urinalysis as a possible entry point for detecting CAKUT before school age [23]. More recently, age-3 urine screening has also been reported to contribute to earlier recognition and treatment consideration in children with hereditary nephropathies such as Alport syndrome [24]. These developments may prompt a reappraisal of the role of age-3 urinalysis, particularly as earlier diagnosis, family screening, and early intervention have become more relevant in selected pediatric kidney diseases [25].

 However, whether the current urine dipstick-based approach is the most appropriate method for these purposes remains uncertain. Dipstick testing for proteinuria and hematuria has limited sensitivity for CAKUT, and diluted urine in young children can lead to false-negative results; therefore, alternative urinary markers and adjunctive renal ultrasonography have been proposed [26–28]. Follow-up after abnormal results are detected has been inconsistent, and final diagnoses often are not well-captured at the municipal level [20]. Therefore, the role of age-3 urinalysis should be evaluated not only by whether urinary abnormalities are detected, but also by whether the screening pathway leads to appropriate confirmatory testing, timely referral, earlier diagnosis, and improved long-term outcomes. Further studies are needed to clarify the optimal screening modalities for potential target conditions, including CAKUT and hereditary nephropathies such as Alport syndrome, and to assess the population-level effectiveness and cost-effectiveness of age-3 urinalysis as a screening program. Before school entry, additional urinalysis opportunities may arise through kindergarten-based screening and, in some municipalities, 4-year-old or 5-year-old health examinations; however, these do not comprise a consistently defined pathway for interpretation and follow-up.

### School age: the school urinary screening program

SUS was introduced in 1974 following revision of the School Health Act and covers students from kindergarten through university within the school health system. With about 15 million students screened annually, SUS is a large national screening program with a historically specific rationale [29, 30]. *JSPN* recommends a positive threshold of proteinuria (≥ 1+) and/or occult blood (≥ 1+) based on the results of a first-morning urine dipstick test; however, practice varies across prefectures, and some programs continue to use lower thresholds or local criteria [23, 31].

The primary target conditions are chronic glomerulonephritis, particularly IgA nephropathy. Long-term data suggest that SUS has mainly detected glomerular disease and may have contributed to the decline in glomerulonephritis-related kidney failure in children and young adults [16, 32]. This historical role remains important. However, the epidemiologic context has changed. CAKUT now accounts for a larger share of pediatric kidney failure than glomerulonephritis, whereas conventional dipstick screening is poorly suited to its detection. Therefore, although SUS remains well aligned with its original purpose of detecting asymptomatic glomerular diseases, urinalysis alone may not fully address the current spectrum of pediatric CKD, particularly CAKUT and other conditions that are not readily detected by dipstick testing [33, 34]. Proposals to add other test components have been discussed, but any expansion would need to be judged not only by detection yield but also by follow-up capacity, clinical benefit, and cost [26, 30].

The economic debate around SUS reflects the same point. Earlier international concerns about urine screening in asymptomatic children focused on uncertain diagnostic yield, false-positive findings, follow-up burden, and cost-effectiveness [35]. However, a recent Japanese analysis found favorable cost-effectiveness for screening targeted to IgA nephropathy [36]. In that model, the assumed cost was ¥200 for screening and ¥18,940 for detailed examination. Sensitivity analyses showed that the result was particularly sensitive to the unit cost of screening; other key drivers included the annual probability of incident detection outside screening and IgA nephropathy incidence. Thus, the favorable result depended not only on the clinical value of early IgA nephropathy detection, but also on the very low cost and existing infrastructure of the Japanese program. A recent review discussed these findings in the broader international debate on pediatric proteinuria screening and suggested that they may offer considerations for other settings, particularly where disease prevalence is underrecognized or access to early nephrology care is limited [37]. Whether SUS remains cost-effective under a broader objective, including conditions such as CAKUT that are less well detected by dipstick urinalysis, remains an open question.

Taken together, SUS is a mature national program with a historically specific rationale and evidence of value for glomerular diseases, especially IgA nephropathy. Its future role in CKD screening will depend on whether it remains focused on its original purpose or is redesigned for the changing spectrum of pediatric kidney disease.

### Adulthood: workplace-based health examinations and the specific health checkup

Urinalysis opportunities in adulthood are provided mainly through workplace-based health examinations and SHC. Neither was originally designed as a CKD screening program, but both have functioned in practice as major starting points of first-line CKD detection in adults.

Workplace-based health examinations were introduced under the Industrial Safety and Health Act for regularly employed workers and are primarily intended for occupational health management [38]. The costs are borne by employers, and both implementation and participation rates are high (91.9% and 81.5%, respectively) [39]. Urinalysis for protein and glucose has long been included as a mandatory component. From April 2027, serum creatinine will be added to the workplace-based health examination panel. However, for workers younger than 40 years, the test may be omitted when a physician judges it unnecessary, on the grounds that kidney dysfunction in this age group is thought to be predominantly accompanied by proteinuria and that blood testing in periodic health examinations is itself subject to physician-based omission rules [13]. Recent modeling studies from the United States suggest that starting CKD screening earlier, including from around age 35 years, may yield greater health benefits than later initiation, although with less favorable cost-effectiveness [40]. In Japan as well, the appropriate age to begin screening, and whether annual testing is necessary, should be re-examined.

SHC, launched in 2008 under the Act on Assurance of Medical Care for Elderly People for adults 40 to 74 years of age, was established in Japan’s medical cost optimization framework as a metabolic syndrome-focused program linked to lifestyle interventions [6]. Although proteinuria testing is included in the basic panel, serum creatinine measurement, and thus eGFR assessment, is not universally included and is performed only under specified conditions. Consequently, participation in the SHC has been incomplete and varies substantially across insurer types (range, 38.2% among those insured by municipality-administered National Health Insurance to 82.9% among those insured by health insurance societies) [41]. Postscreening follow-up is also limited. Among individuals with CKD that was newly identified by the SHC, only a limited proportion seek medical care after abnormal results are detected [42]. At the same time, higher prefecture-level SHC participation has been reported to be associated with lower incidence of treated kidney failure [43].

Considered together, these programs reveal three structural challenges for CKD screening in adulthood. First, screening pathways remain fragmented by employment status, age, and insurance arrangements, with no uniform national pathway, especially for adolescents and young adults (AYA, typically aged 15 -39 years). Second, a urine dipstick test cannot detect CKD in the absence of proteinuria. This limitation is particularly relevant for adult CKD that may present with little or no proteinuria, including nephrosclerosis or ischemic nephropathy, some hereditary kidney diseases such as autosomal dominant polycystic kidney disease or autosomal dominant tubulointerstitial kidney disease, and drug-induced kidney injury. Even after the addition of serum creatinine in workplace-based health examinations, this gap remains for adults younger than 40 years and those without workplace-based screening opportunities. Third, important limitations beyond test selection exist, including incomplete follow-up after abnormal findings are detected. Cost-effectiveness analyses predate recent policy and therapeutic changes; therefore, updated analyses under the current conditions are necessary [44, 45].

### Older adults: late-stage elderly medical examinations

At age 75, individuals transition to the late-stage elderly medical care system, which is administered by prefectural-level wide-area associations under the Act on Assurance of Medical Care for Elderly People. Health examinations under this system are conducted on a best-efforts basis rather than as a statutory obligation [46]. The latest publicly reported participation rate was 28.1%, below the 30% benchmark used in the insurer incentive framework [47]. Serum creatinine is generally treated as an additional test [48]. Even when serum creatinine is measured, interpretation of creatinine-based eGFR in this age group may be complicated by reduced muscle mass, and the possible role of cystatin C in selected older adults requires further consideration [49]. The role of urinalysis in this setting as CKD screening is less clear: more than 95% of beneficiaries have at least one outpatient visit each year, with nearly half of outpatient users having visits every month, and neither screening positivity nor renal outcomes having been clearly defined for this population [50].

### International context

 Internationally, Japan is distinctive but not isolated. Some East Asian settings, for example Korea and Taiwan, have also incorporated kidney-related testing into population-based health examination systems, including school urinary screening and adult health checkups with urine and kidney function tests [51–54]. Several Western recommendations have traditionally been cautious about routine urine screening in asymptomatic children and about untargeted CKD screening in asymptomatic adults [4, 35, 55, 56]. However, this distinction is becoming less absolute. The USPSTF is currently updating its CKD screening recommendation, and recent cost-effectiveness analyses from the United States suggest that population-wide albuminuria screening may be economically attractive in the era of SGLT2 inhibitors, particularly from middle age onward [40, 57, 58] A recent comprehensive review similarly identified albuminuria as a key marker for CKD screening and risk stratification and noted that population screening strategies using eGFR and albuminuria are becoming increasingly cost-effective with the availability of novel therapies [49]. These results were sensitive to assumptions about SGLT2 inhibitor effectiveness and cost, as well as screening frequency and age at initiation. Therefore, the value of population-based CKD screening depends not only on test performance, but also on the availability, cost, and effectiveness of downstream treatment. Japan’s system should thus be understood within a broader international debate over when population-based kidney screening is justified. Its main challenge is to align existing testing opportunities with target conditions, follow-up pathways, and cost-effectiveness.

## Challenges and future directions

This review identifies four structural challenges: mismatch between screening tests and target conditions, limitations in the pathway from detection to care, lack of continuity in kidney-related information across programs, and limited evidence to guide policy. First, the match between tests and target conditions is often poor. In childhood, dipstick testing has limited sensitivity for CAKUT, although how CAKUT should be incorporated into existing screening programs remains under discussion. In adulthood, dipstick urinalysis misses CKD without proteinuria, and the addition of serum creatinine addresses this problem only partly, among adults aged 40 years and older who are covered by workplace-based health examinations. In the late-stage elderly, the role of urinalysis in CKD screening remains uncertain. Table 1 summarizes current and potential target conditions and remaining questions for each life-stage program. This table is not intended to define fixed screening targets, but to clarify where current modalities are well aligned with candidate conditions and where further policy evaluation is needed. Second, the pathway from detection to care is limited. Abnormal screening results do not necessarily lead to confirmatory testing, guideline-based risk stratification, medical consultation, or referral when indicated [42, 59, 60]. This is not unique to Japan. Globally, CKD care is characterized by a gap between detection and implementation: many people with CKD remain undiagnosed, and even after diagnosis, appropriate treatment and referral are not consistently delivered [61]. Because evidence has indicated that collaborative CKD care can improve outcomes, better follow-up systems may improve screening effectiveness [62]. Third, kidney-related information is not consistently carried forward across health examination programs. Japan’s life-course health examinations are not longitudinally linked at the individual level, partly because they are administered under different legal frameworks and by different responsible bodies. Recent policy initiatives have begun to promote digital access to health examination data, but these efforts remain at an early stage and are not yet organized around kidney-specific screening pathways [63]. In the future, longitudinal management of health examination data could support risk-stratified alerts based on earlier kidney-related findings and help guide test selection, referral, and follow-up in later screening opportunities. Fourth, policy-relevant evidence remains limited. Screening requires evidence that earlier detection improves outcomes compared with usual clinical detection, that benefits outweigh harms such as false-positive results, labeling effects, and overdiagnosis, and that the approach is justified in terms of cost-effectiveness. Japan’s existing programs have not yet been sufficiently evaluated in these respects.

**Table 1:** Current and potential target conditions and remaining questions for kidney screening across life-course health examination systems in Japan

Life stage	Current and potential target conditions	Remaining questions
Early childhood	CAKUT; hereditary nephropathies such as Alport syndrome	Should CAKUT, Alport syndrome, or other hereditary nephropathies be explicit targets of age-3 screening? Which modalities, including urine dipstick testing, urinary markers, ultrasonography, or targeted genetic evaluation in selected cases, are most appropriate? Is age-3 urinalysis effective and cost-effective as a population-based screening program?
School age	Persistent urinary abnormalities; chronic glomerulonephritis, particularly IgA nephropathy	Should SUS remain focused on proteinuric glomerular disease, or should it be redesigned to address the broader spectrum of pediatric CKD? If additional components are introduced, how should detection yield, follow-up capacity, clinical benefit, and cost be assessed?
Working-age adults under 40 years	Proteinuric CKD; non-proteinuric CKD identifiable by reduced eGFR if serum creatinine is measured; CKD associated with hypertension, diabetes, obesity, or other cardiometabolic risk factors	How should the planned 2027 implementation of serum creatinine to workplace-based health examinations be operationalized, particularly given age-based omission rules and gaps among people outside regular employment? How should abnormal eGFR or urinalysis results be linked to confirmatory testing and medical care?
Midlife and older adults, ages 40–74 years	Diabetic kidney disease; hypertensive nephrosclerosis; proteinuric or albuminuric CKD; reduced eGFR; CKD associated with cardiometabolic risk	How should existing CKD risk-stratification and referral criteria based on eGFR and proteinuria or albuminuria be implemented within the SHC pathway? Should UACR be incorporated more consistently for CKD and cardiovascular risk stratification, especially among people with diabetes, hypertension, or other cardiometabolic risks?
Late-stage, ages 75 years and older	Reduced eGFR; proteinuric or albuminuric CKD; CKD associated with multimorbidity, frailty, hypertension, diabetes, or cardiovascular disease	How should existing CKD risk-stratification and referral criteria be applied while accounting for age-related eGFR decline, multimorbidity, frailty, competing risks, and patient goals? How might cystatin C be used in selected older adults to improve GFR estimation and risk prediction?

When viewed in the context of its historical development, Japan’s urine-based screening system—despite its limitations—represents a public health asset accumulated over decades. SUS, together with JSPN guidance and local implementation efforts, has built an evidence base and operational framework for pediatric kidney disease screening. In adult CKD screening, JSN has achieved an important policy change through sustained advocacy for adding serum creatinine to workplace-based health examinations. The next step is to link these achievements rather than replace the existing systems. A practical strategy would specify, for each program, the target conditions, appropriate test components, actions following abnormal findings, and mechanisms for carrying kidney-related information forward—thereby transforming repeated but isolated testing opportunities into a coherent screening-to-care pathway across the life course. Implementing such a strategy will require sustained collaboration among academic societies, public health authorities, insurers, health examination providers, and clinicians.

## Conclusion

Japan’s health examination systems provide urinalysis opportunities across the life course; however, their accumulation alone does not ensure a coherent CKD screening strategy. Gaps remain in case detection, follow-up, continuity of kidney-related information, and policy-relevant evidence. The addition of serum creatinine to workplace-based health examinations, beginning in 2027, marks an important turning point, showing that accumulated evidence can lead to policy change. Building a more connected CKD screening strategy across the life course will require clearer decisions at each stage about which conditions to target, how they should be detected, how abnormal findings should lead to follow-up, and which outcomes should be used to evaluate program value.
